# Analysis of α-dicarbonyl compounds and volatiles formed in Maillard reaction model systems

**DOI:** 10.1038/s41598-019-41824-8

**Published:** 2019-03-29

**Authors:** Jiyoon Cha, Trishna Debnath, Kwang-Geun Lee

**Affiliations:** Department of Food Science and Biotechnology, Dongguk University-Seoul, 32, Dongguk-ro, Ilsandong-gu, Goyang-si, Gyeonggi-do 410-820 Republic of Korea

## Abstract

In this study, production of three α-dicarbonyl compounds (α-DCs) including glyoxal (GO), methylglyoxal (MGO), and diacetyl (DA) as well as volatile flavor compounds was analyzed using Maillard reaction (MR) model systems. A total of 16 model systems were assembled using four amino acids and four reducing sugars, and reactions were performed at 160 °C and pH 9. Determination of α-DCs was conducted using a gas chromatography/nitrogen phosphorous detector (GC-NPD) after derivatization and liquid-liquid extraction. α-DC levels in MR model systems were 5.92 to 39.10 μg/mL of GO, 3.66 to 151.88 μg/ml of MGO, and 1.10 to 6.12 μg/mL of DA. The highest concentration of total α-DCs was found in the fructose-threonine model system and the lowest concentration in the lactose-cysteine model system. Volatile flavor compounds were analyzed using solid-phase micro-extraction (SPME) followed by GC-mass spectrometry (GC-MS). Different volatile flavor compound profiles were identified in the different MR model systems. Higher concentrations of α-DCs and volatile flavor compounds were observed in monosaccharide-amino acid MR model systems compared with disaccharide-amino acid model systems.

## Introduction

The Maillard reaction is the most basic chemical reaction occurring in many foods, in the form of a non-enzymatic browning reaction between a reducing sugar and an amino acid. Maillard reaction products (MRPs) generally have a positive impact on taste, color, and flavor of food, but some of them can have a negative toxic impact as well.

α-Dicarbonyl compounds (α-DCs) are yellow colored, low molecular weight organic compounds containing two carbonyl groups on the α-carbon^1^. They are formed from sugar fragmentation during non-enzymatic browning and are intermediates in caramelization and the Maillard reaction^2^, they are also formed during oxidative degradation. In addition, α-DCs exist in fermented foods and beverages. Approximately 18 kinds of α-DCs have been identified in various foods, among which glyoxal (GO), methylglyoxal (MGO), and diacetyl (DA) are the most representative. Recent studies have found that α-DCs can pose a safety risk^3^. Amoroso *et al*.^3^ reported the cytotoxic effect of α-DCs *in vitro*^3^. According to their results, both undigested and digested α-DCs induce cytotoxicity in human cells and inhibit human DNA repair enzymes. Further, Morgan *et al*.^4^ reported respiratory toxicity of diacetyl in C57Bl/6 mice reproducing characteristics of human obliterative bronchiolitis. In addition, α-DCs were reported as precursors of toxic substances such as 4(5)-methylimidazole, heterocyclic flavor compounds with respiratory toxicity^5^. α-DCs react with free amino group of proteins to form the Advanced glycation endproducts (AGEs) which associated with diabetes and kidney disease in the human body^6^. It has been reported that inhalation of glyoxal causes local irritation of the eyes and respiratory organs. Oral exposure to glyoxal may also cause congestion of gastrointestinal tract, lung, and kidney^7^. In this study, α-DCs were analyzed as potential undesirable products of the Maillard reaction.

Previous studies have reported α-DC analysis through derivatization to quinoxalines by o-phenylenediamine (OPD) to terminate the Maillard reaction^8,9^. This reaction easily occurs at room temperature in a slightly basic solution^10^, and allows detection using various analytical methods including liquid chromatography, gas chromatography-mass spectrometry, and gas chromatography-nitrogen phosphorous detection^11^.

Aroma compounds in Maillard reactions are formed based on types of sugars and amino acids present, temperature, reaction time, and pH^12^. Over 100 types of such aroma compounds have been found in conventional flavor studies. Among them, roasted and sweet flavors are accepted as having positive effects in foods such as coffee, caramel sauce, and bread. Pyrazines, representative compounds of the roasted flavor, are formed by reaction of amines and α-dicarbonyls through Strecker degradation^13,14^. Formation of furaneols, representative compounds of the sweet flavor, occurs through the 2, 3-enolization pathway leading to 1-deoxyosones as intermediates^15^.

MRPs that consist mainly of low-molecular volatile compounds are difficult to sample and analyze, and individual compound analysis has been conducted in such cases. Studies of MRPs with sensory characteristics have also been performed. However, studies comparing desirable aroma compounds and undesirable toxic compounds are not common. In this study, we compared α-dicarbonyl compounds and aroma compounds produced in reducing sugar–amino acid model systems. This work puts down the bases for studies on desirable preference factor like flavors and undesirable toxic substances.

## Materials and Methods

### Chemicals

D-Glucose, D-fructose, D-maltose, D-lactose, L-lysine, L-serine, L-threonine, L-cysteine, L-norvaline, glyoxal, methylglyoxal, diacetyl, quinoxaline, 2-methylquinoxaline, 2,3-dimethylquinoxaline, o-phenylenediamine dihydrochloride, 1-methylpyrazole, C8-C20 alkane standard, methyl cinnamate, and sodium chloride were purchased from Sigma-Aldrich (St. Louis, MO). Water and ethyl acetate were obtained from J.T. Baker (Philipsburg, NJ, USA). All solutions were stored at 4 °C until analysis. Divinylbenzene/Carboxen/Polydimethylsiloxane (DVB/CAR/PDMS, 50 μm film thickness) solid phase microextraction (SPME) fiber, polypropylene hold caps, and polytetrafluoroethylene (PTFE)/silicone septa were purchased from Supelco Inc. (Belfonte, PA, USA).

### Preparation of Maillard reaction model system solutions

Equimolar solutions (0.1 M) of four reducing sugars (glucose, fructose, lactose, and maltose) and four amino acids (lysine, serine, threonine, and cysteine) were prepared in distilled water and pH was set to 9. Solutions were placed in swing-top bottles and reactions were performed at 160 °C for 2 h in an oven (OF-22, Jeiotech Co., Seoul, Korea) to instigate the Maillard reaction. All solutions were cooled under running cold water to room temperature immediately in order to stop the reaction progress. All samples were stored at 4 °C until they were analyzed.

### Analysis of α-Dicarbonyl compounds

Quantification and analysis of α-DCs produced in the Maillard reaction model systems was performed using o-phenylenediamine dihydrochloride derivatization reported in a previous study^1^. A 3 mL volume of each sample and 2 mL of o-phenylenediamine dihydrochloride were placed in 20 mL vials. The mixtures were set to pH 12 and stirred for 2 h at 600 rpm for derivatization of α-DCs to quinoxalines. Then, 5 mL of ethyl acetate was added to the mixtures and shaken for extraction. Extracted solutions were analyzed using GC-NPD. The concentrations used to perform standard calibration curve were 0.5, 1, 5, 10, 50, 100 µg/ml.

An Agilent 6890 N gas chromatograph with a nitrogen phosphorous detector was used for analysis of α-DCs. A DB-WAX column (30 m × 250 × 0.25 μm; J&W Scientific, Folsom, CA) was used for separation. Helium was used as carrier gas at constant flow of 1.5 mL/min. Injection was set to the splitless mode at 260 °C. Oven temperature was held at 40 °C for 2 min and then increased to 170 °C at 20 °C/min and held for 15 min. Detector temperature was set at 300 °C. Nitrogen was used as make-up gas at a flow rate of 5 mL/min.

### Analysis of aroma compounds

Aroma compounds were analyzed using SPME (Solid phase micro-extration). A 5-mL volume of sample was added to a 20 mL headspace vial containing 0.75 g of sodium chloride. 5 μL of methyl cinnamate (internal standard, 100 μg/mL), 10 μL of alkane standard (10 μg/mL), and a magnetic stirring bar were added and the vial sealed with a PTFE cap. Subsequently, samples were stirred for 30 min at 70 °C to reach equilibrium. Volatile flavor compound extraction was carried out by injecting a fiber into the vial for adsorption at 70 °C for 10 min. Then, the fiber was inserted into the GC injector port and held for 10 min to desorb the volatile flavor compounds.

An Agilent 7820 A gas chromatograph and a 5977E mass spectrometer were used for volatile flavor compound detection. Separation of volatile aroma compounds was performed using a DB-WAX column (60 m × 250 × 0.25 μm). The oven was held at 40 °C for 5 min, then raised to 185 °C at 5 °C/min and held for 20 min, and then raised to 200 °C at 10 °C/min and held for 5 min. Helium was used as carrier gas at a constant flow rate of 0.8 mL/min. Injection mode was set to splitless at 230 °C. The detector was set to the scan mode and the scan range was from 20 to 550 m/z. Volatile flavor compounds were identified based on Kovats index on DB-WAX, co-injection, and mass spectrum from the NIST library. The quantification of each flavor compound was displayed as peak area ratio (PAR, peak area of each compound/that of internal standard).

### Statistical analysis

All samples were analyzed in triplicate and the analysis results are presented as mean ± standard deviation.

## Results and Discussion

### Analysis of α-DCs in Maillard reaction model systems

Reaction model solutions were prepared with glucose and lysine at four temperatures (100, 120, 140 and 160 °C) and four pH levels (3,5,7, and 9) to simulate Maillard reaction model system conditions. Level of different α-DCs produced in the reactions, as shown in Table 1 was 0.36 to 16.78 μg/mL of GO, trace to 50.81 μg/mL of MGO, and N.D. to 2.27 μg/mL of DA. Higher pH and temperature resulted in greater production of total α-DCs including GO, MGO, and DA. Conditions yielding the maximum concentration of total α-DCs were 160 °C and pH 9. These conditions were then used to study the reducing sugar-amino acid model systems. As shown in Table 2, a total of 16 model systems were used with combinations of four amino acids (lysine, serine, threonine, and cysteine) and four reducing sugars (glucose, fructose, maltose, and lactose). Under the above conditions, basic amino acids containing hydroxyl groups have higher reactivity with α-DCs than acidic and nonpolar amino acids^16^. Levels of α-DCs in MR model systems were 5.92 to 39.10 μg/mL of GO, 3.66 to 151.88 μg/mL of MGO, and 1.10 to 6.12 μg/mL of DA. The highest concentration of α-DCs was detected in the fructose-threonine model system and the lowest concentration in the lactose-cysteine model system. In addition, DA concentration was 1.66 to 6.12 μg/mL in lactose model systems, compared with other reducing sugar model systems. These results indicated that α-DCs were produced mainly from monosaccharides rather than from disaccharides. Hollnagel and Kroh reported that monosaccharides forms more α-DCs than disaccharides^2^. Different from our results among monosaccharides glucose formed more α-DCs than fructose^2^. In the report they suggested that fructose forms more cyclic compounds rather than fragmentation products such as α-DCs. However, Maillard reaction conditions were different and it could give rise to the conflicting results.

**Table 1:** Concentrations (μg/mL) of glyoxal (GO), methylglyoxal (MGO), and diacetyl (DA) produced in the glucose–lysine Maillard reaction model system.

Sample	GO	MGO	DA
100 °C- pH 3	0.36 ± 0.04	trace	0.45 ± 0.01
100 °C- pH 5	0.45 ± 0.01	0.18 ± 0.01	N.D.
100 °C- pH 7	0.55 ± 0.01	0.27 ± 0.01	N.D.
100 °C- pH 9	0.67 ± 0.04	0.12 ± 0.01	0.39 ± 0.01
120 °C- pH 3	1.10 ± 0.06	0.59 ± 0.05	0.68 ± 0.01
120 °C- pH 5	0.86 ± 0.02	0.12 ± 0.00	0.71 ± 0.03
120 °C- pH 7	0.97 ± 0.01	0.11 ± 0.01	0.76 ± 0.03
120 °C- pH 9	3.36 ± 0.10	2.41 ± 0.02	0.81 ± 0.04
140 °C- pH 3	2.03 ± 0.07	1.85 ± 0.10	0.78 ± 0.02
140 °C- pH 5	1.73 ± 0.06	0.69 ± 0.07	0.67 ± 0.02
140 °C- pH 7	1.92 ± 0.04	0.31 ± 0.02	0.86 ± 0.01
140 °C- pH 9	16.78 ± 0.18	15.62 ± 0.62	1.41 ± 0.03
160 °C- pH 3	2.27 ± 0.10	0.62 ± 0.05	0.50 ± 0.02
160 °C- pH 5	11.93 ± 0.09	3.55 ± 0.04	0.36 ± 0.01
160 °C- pH 7	15.79 ± 0.38	5.79 ± 0.20	0.41 ± 0.01
160 °C- pH 9	11.81 ± 0.19	50.81 ± 0.59	2.27 ± 0.03

**Table 2:** Concentrations (μg/mL) of glyoxal (GO), methylglyoxal (MGO), and diacetyl (DA) produced in reducing sugar and amino acid Maillard reaction model systems at 160 °C and pH 9.

Sample	GO	MGO	DA
Glu + Lys	11.81 ± 0.19	50.81 ± 0.60	2.27 ± 0.03
Glu + Ser	34.31 ± 2.00	79.04 ± 1.75	3.09 ± 0.09
Glu + Thr	18.82 ± 0.70	54.79 ± 1.47	1.79 ± 0.05
Glu + Cys	8.92 ± 0.32	24.42 ± 0.52	1.43 ± 0.02
Fru + Lys	35.42 ± 0.10	67.86 ± 0.66	1.57 ± 0.03
Fru + Ser	39.10 ± 0.16	106.53 ± 1.48	3.12 ± 0.10
Fru + Thr	10.93 ± 0.32	151.88 ± 1.43	3.29 ± 0.10
Fru + Cys	7.34 ± 0.61	27.00 ± 1.40	1.10 ± 0.02
Mal + Lys	29.30 ± 0.30	6.41 ± 0.22	1.32 ± 0.03
Mal + Ser	24.09 ± 0.88	12.69 ± 0.62	1.75 ± 0.04
Mal + Thr	17.10 ± 0.30	57.58 ± 1.27	3.88 ± 0.20
Mal + Cys	5.96 ± 0.19	4.92 ± 0.12	1.49 ± 0.04
Lac + Lys	24.47 ± 0.20	8.90 ± 0.09	6.12 ± 0.00
Lac + Ser	27.00 ± 0.67	34.04 ± 1.65	5.34 ± 0.17
Lac + Thr	12.62 ± 0.29	36.10 ± 0.88	3.49 ± 0.09
Lac + Cys	5.92 ± 0.27	3.66 ± 0.14	1.66 ± 0.05

### Aroma compounds formed in Maillard reaction model systems

Volatile flavor compounds formed in the reaction model systems were identified. Results of analysis showed that volatile flavor compounds were primarily composed of eight compounds representing roasted flavor (2,3-dimethyl pyrazine, 2,5-dimethyl pyrazine, 2,6- dimethyl pyrazine, 2,3,5-trimethyl pyrazine, 2-ethyl-3,6-dimethyl pyrazine, 2-ethyl-3,5- dimethyl pyrazine, 2,3-dimethyl-5-methylpyrazine, and 2-Acetylthiazole) and five representing sweet flavor (benzaldehyde, 5-methylfurfural, furaneol, homofyraneol, and norfuraneol).

Table 3 shows results from glucose (Glu)–amino acid (lysine, serine, threonine and cysteine) model systems. Total peak area ratio (PAR) in Glu–Lys, Glu–Ser, Glu–Thr and Glu–Cys model systems was 1.3606, 0.7369, 1.5351, and 0.2322, respectively. The compound 2,5-dimethyl pyrazine representing odor of chocolate and roasted nuts was detected significantly in Glu–-Lys, Glu–Ser, Glu–Thr, and Glu–Cys model systems with PAR of 1.0248, 0.4728, 0.6512, and 0.0692, respectively^17^. The PARs of 2,5-dimethyl pyrazine were 26–75% in the model systems compared to total PAR. In the previous study 2, 5-dimethyl pyrazine was reported as a main flavor in Maillard reaction model systems^13^.

**Table 3:** Aroma compounds produced in glucose (Glu)–amino acid (lysine (Lys), serine (Ser), threonine (Thr), and cysteine (Cys)) Maillard reaction model systems.

Compounds	Retention time	K.I	K.I (Ref)	Co-injection	PAR (peak area ratio)			
					Glu + Lys	Glu + Ser	Glu + Thr	Glu + Cys
2,3-Dimethyl pyrazine	25.609	1372	1375	o	0.0295 ± 0.0035	N.D	N.D	0.0093 ± 0.0009
2,5-Dimethyl pyrazine	24.878	1363	1350	o	1.0248 ± 0.1526	0.4728 ± 0.0176	0.6512 ± 0.0559	0.0692 ± 0.0068
2,6-Dimethyl pyrazine	25.041	1356	1356	o	0.0370 ± 0.0054	0.0229 ± 0.0006	0.0143 ± 0.0006	0.1052 ± 0.0106
2,3,5-Trimethyl pyrazine	27.270	1434	1430	o	0.0964 ± 0.0093	0.0817 ± 0.0043	0.1174 ± 0.0099	0.0109 ± 0.0009
2-Ethyl-3,6-dimethylpyrazine	28.400	1474	1474	o	0.1590 ± 0.0154	0.1907 ± 0.0033	0.7304 ± 0.0600	0.0030 ± 0.0003
2-Ethyl-3,5-dimethylpyrazine	28.829	1490	1491	o	N.D	0.0073 ± 0.0004	0.0159 ± 0.0015	N.D
2-Acetylthiazole	33.705	1686	1686	o	N.D	N.D	N.D	0.0223 ± 0.0022
Benzaldehyde	30.642	1562	1562	o	0.0140 ± 0.0020	0.0064 ± 0.0003	0.0059 ± 0.0002	0.0124 ± 0.0009

In the Glu–Thr model system, 2-ethyl-3,5-dimethylpyrazine was the highest detected with peak area ratio 0.7304. In addition, 2-acetylthiazole representing popcorn-like^18^ flavor had a peak area ratio of 0.0223 in the Glu–Cys sulfur model system. Benzaldehyde, representing sweet burnt sugar and roasted almond flavors^19^ had a peak area ratio of 0.0140, 0.0064, 0.0059, and 0.0124, respectively in Glu–Lys, Glu–Ser, Glu–Thr, and Glu–Cys model systems. 2,3-Dimethyl-5-methylpyrazine, representing roasted flavor and 5-Methylfurfural, Furaneol, Homofuraneol, and Norfuraneol representing sweet flavor were not detected.

Table 4 shows results from fructose (Fru)–amino acid (Lys, Ser, Thr, and Cys) model systems. Total peak area ratio in Fru–Lys, Fru–Ser, Fru–Thr, and Fru–Cys model systems was 1.1592, 0.7983, 2.6741, and 0.1341, respectively. The compound 2,5-Dimethyl pyrazine was detected with peak area ratio of 0.9241, 0.4980, 1.0404, and 0.0396, respectively in Fru–Lys, Fru–Ser, Fru–Thr, and Fru–Cys model systems. In the Fru–Cys model system, 2, 6-dimethyl pyrazine was the highest detected with peak area ratio 0.0567. In addition, 2-acetylthiazole showed a peak area ratio of 0.0140 in the Fru–Cys model system. The compounds 2,3-Dimethyl-5-methylpyrazine, 5-Methylfurfural, Furaneol, Homofuraneol, and Norfuraneol were not detected.

**Table 4:** Aroma compounds produced in fructose (Fru)–amino acid (lysine (Lys), serine (Ser), threonine (Thr), and cysteine (Cys)) Maillard reaction model systems.

Compounds	Retention time (min)	K.I	K.I (Ref)	Co-injection	PAR (peak area ratio)			
					Fru + Lys	Fru + Ser	Fru + Thr	Fru + Cys
2,3-Dimethyl pyrazine	25.609	1372	1375	o	0.0216 ± 0.0140	N.D	N.D	0.0024 ± 0.0001
2,5-Dimethyl pyrazine	24.878	1363	1350	o	0.9241 ± 0.4840	0.4980 ± 0.0129	1.0404 ± 0.0401	0.0396 ± 0.0011
2,6-Dimethyl pyrazine	25.041	1356	1356	o	0.0211 ± 0.0100	0.0298 ± 0.0009	0.0635 ± 0.0025	0.0567 ± 0.0015
2,3,5-Trimethyl pyrazine	27.270	1434	1430	o	0.0564 ± 0.0370	0.1060 ± 0.0034	0.4718 ± 0.0159	0.0043 ± 0.0000
2-Ethyl-3,6-dimethylpyrazine	28.400	1474	1474	o	0.1326 ± 0.059	0.1522 ± 0.0055	1.0390 ± 0.0309	0.0017 ± 0.0000
2-Ethyl-3,5-dimethylpyrazine	28.829	1490	1491	o	N.D	0.0084 ± 0.0002	0.0560 ± 0.0018	N.D
2-Acetylthiazole	33.705	1686	1686	o	N.D	N.D	N.D	0.0140 ± 0.0006
Benzaldehyde	30.642	1562	1562	o	0.0035 ± 0.0001	0.0038 ± 0.0003	0.0034 ± 0.0001	0.0155 ± 0.0003

Table 5 shows results from maltose (Mal)–amino acid (Lys, Ser, Thr, and Cys) model systems. Total peak area ratio in Mal–Lys, Mal–Ser, Mal–Thr and Mal–Cys model systems was 0.0066, 0.0161, 0.0217, and 0.0110, respectively. Benzaldehyde was detected with peak area ratio of 0.0041, 0.0051, 0.0053, and 0.0110, respectively in Mal–Lys, Mal–Ser, Mal–Thr, and Mal–Cys model systems. The compounds 2,3-dimethyl pyrazine, 2,6-dimethyl pyrazine, 2-ethyl-3,5-dimethylpyrazine, 2,3-Dimethyl-5-methylpyrazine, 2-acethylthiazole, 5-methylfurfural, Furaneol, Homofuraneol, and Norfuraneol were not detected.

**Table 5:** Aroma compounds produced in maltose (Mal)–amino acid (lysine (Lys), serine (Ser), threonine (Thr), and cysteine (Cys)) Maillard reaction model systems.

Compounds	Retention time	K.I	K.I (Ref)	Co-injection	PAR (peak area ratio)			
					Mal + Lys	Mal + Ser	Mal + Thr	Mal + Cys
2,3-Dimethyl pyrazine	25.609	1372	1375	o	N.D	N.D	N.D	N.D
2,5-Dimethyl pyrazine	24.878	1363	1350	o	0.0024 ± 0.0002	0.0046 ± 0.0005	0.0053 ± 0.0002	N.D
2,6-Dimethyl pyrazine	25.041	1356	1356	o	N.D	N.D	N.D	N.D
2,3,5-Trimethyl pyrazine	27.270	1434	1430	o	N.D	0.0064 ± 0.0005	0.0063 ± 0.0002	N.D
2-Ethyl-3,6-dimethylpyrazine	28.400	1474	1474	o	N.D	N.D	0.0047 ± 0.0000	N.D
2-Ethyl-3,5-dimethylpyrazine	28.829	1490	1491	o	N.D	N.D	N.D	N.D
2-Acetylthiazole	33.705	1686	1686	o	N.D	N.D	N.D	N.D
Benzaldehyde	30.642	1562	1562	o	0.0041 ± 0.0004	0.0051 ± 0.0003	0.0053 ± 0.0004	0.0110 ± 0.0006

Table 6 shows results from lactose (Lac)–amino acid (Lys, Ser, Thr, and Cys) model systems. Total peak area ratio in Lac–Lys, Lac–Ser, Lac–Thr, and Lac–Cys model systems was 0.0053, 0.0063, 0.5224, and 0.0043, respectively. Benzaldehyde was detected with peak area ratio of 0.0053, 0.0063, 0.0043, and 0.0043, respectively in Lac– Lys, Lac–Ser, Lac–Thr, and Lac–Cys model systems. In the Lac–Thr model system, 2,5-dimethyl pyrazine, 2,3,5-trimetyl pyrazine, 2-ethyl-3,6-dimethylpyrazine, and 2-ethyl-3,5-dimethylpyrazine were present with peak area of 0.0070, 0.04961, 0.0086, and 0.0064, respectively. The compounds 2, 3-dimethyl pyrazine, 2,6-dimethyl pyrazine, 2,3-Dimethyl-5-methylpyrazine, 2-acethylthiazole, 5-Methylfurfural, Furaneol, Homofuraneol, and Norfuraneol were not detected. Overall, in the reducing sugar and amino acid Maillard reaction model systems, roasted flavors were produced at higher levels while sweet flavor was produced at lesser levels.

**Table 6:** Aroma compounds produced in lactose (Lac)–amino acid (lysine (Lys), serine (Ser), threonine (Thr), and cysteine (Cys)) Maillard reaction model systems.

Compounds	Retention time	K.I	K.I (Ref)	Co-injection	PAR (peak area ratio)			
					Lac + Lys	Lac + Ser	Lac + Thr	Lac + Cys
2,3-Dimethyl pyrazine	25.609	1372	1375	o	N.D	N.D	N.D	N.D
2,5-Dimethyl pyrazine	24.878	1363	1350	o	N.D	N.D	0.0070 ± 0.0004	N.D
2,6-Dimethyl pyrazine	25.041	1356	1356	o	N.D	N.D	N.D	N.D
2,3,5-Trimethyl pyrazine	27.270	1434	1430	o	N.D	N.D	0.4961 ± 0.0310	N.D
2-Ethyl-3,6-dimethylpyrazine	28.400	1474	1474	o	N.D	N.D	0.0086 ± 0.0004	N.D
2-Ethyl-3,5-dimethylpyrazine	28.829	1490	1491	o	N.D	N.D	0.0064 ± 0.0005	N.D
2-Acetylthiazole	33.705	1686	1686	o	N.D	N.D	N.D	N.D
Benzaldehyde	30.642	1562	1562	o	0.0053 ± 0.0004	0.0063 ± 0.0002	0.0043 ± 0.0004	0.0043 ± 0.0004

## Conclusions

In this study, desirable volatile flavor compounds and undesirable α-dicarbonyl compounds formed during the Maillard reaction were analyzed using reducing sugar–amino acid model systems. The levels of α-DCs produced in MR model systems were 1.10 to 151.88 μg/mL. The highest concentration of total α-DCs was found in the fructose–threonine model system and the lowest concentration of total α-DCs was found in the lactose–cysteine model system. Different volatile flavor compound profiles were identified from different MR model systems. Higher concentrations of α-DCs and volatile flavor compounds were observed in monosaccharide–amino acid Maillard model systems than in disaccharide–amino acid model systems. As a future work, the balanced study between desirable flavor compounds and toxic compounds such as α-DCs are supposed to be carried out in our laboratory.
